# Pathogenesis, Assessments, and Management of Chemotherapy-Related Cognitive Impairment (CRCI): An Updated Literature Review

**DOI:** 10.1155/2020/3942439

**Published:** 2020-06-24

**Authors:** Longqin Lv, Shanping Mao, Huimin Dong, Pan Hu, Ruochen Dong

**Affiliations:** Department of Neurology, Renmin Hospital of Wuhan University, Wuhan, Hubei 430060, China

## Abstract

There are various cancer treatments at present, and chemotherapy is one of the main methods. Chemotherapy-related cognitive impairment (CRCI), as one of the side effects of chemotherapy, has gradually attracted the attention of more and more researchers. CRCI has been verified by subjective reports and objective neuropsychological tests so far. But oncologists' understanding of it and its treatments are still incomplete. In this review, we mainly give a comprehensive overview of the mechanism of CRCI, then describe a variety of evaluation methods, and finally summarize the treatment approaches under current medical conditions and compare it with an excellent article published in 2015 with the aim of providing directions for future research and better understanding of CRCI for clinicians.

## 1. Introduction

The number of survivors with cancer in China is giant. Clinicians previously focused on cancer-specific clinical endpoints, such as overall survival, progression-free survival, and objective response rate. With the improvement of scientific and technological level, cancer diagnosis and treatment technology has been well developed, the survival rate of patients with cancer has increased, and the mortality rate has decreased. Therefore, the quality of life of patients is receiving more and more attention. As an important component of quality of life, cognitive function decline in cancer patients has been reported in numerous studies [1–4].

Silberfarb first introduced the concept of chemotherapy-related cognitive impairment (CRCI) in 1983 [5]. Since then, more and more researchers have found its existence in different diseases [6–9]. CRCI, also known as chemobrain or chemofog, can occur in many cancer patients, such as breast cancer, lung cancer, leukemia, lymphoma, colorectal cancer, ovarian cancer, prostate cancer, and testicular cancer. CRCI is usually mild to moderate, and individuals mainly show defects in the cognitive domain including memory, attention, executive function, vocabulary fluency, processing speed, and response time after chemotherapy. In fact, CRCI is more appropriately defined in terms of cancer-associated cognitive impairment, as some studies have also found cognitive impairment in cancer patients without any treatment [10]. However, not all cancer patients develop CRCI after chemotherapy. The incidence of CRCI is about 17% to 70%, and it has even reached 75% in breast cancer [11]. Cognitive impairment can occur during or shortly after chemotherapy, or late CRCI can occur late in the course of the disease. CRCI may exist for a long time [12], but some studies have also found that cognitive disorders show a trend of improvement over time [13]. The specific cause of this difference is still unknown and may be related to the patients' cognitive status at baseline. In 2015, Wefel et al. published an excellent review that comprehensively described the clinical characteristics, pathophysiology, and management of the non-central nervous system CRCI in adults [11]. As time went by, the researchers made more wonderful discoveries. So, our literature review tries to explore new and unique contribution from them.

## 2. Pathogenesis

### 2.1. Direct Neurotoxic Effects of Chemotherapy Drugs

Chemotherapy may damage the brain metabolism and cellular function through direct cytotoxic damage to nerve cells. Platinum-based chemotherapy is one of the currently widely used antitumor regimens, including lung cancer, ovarian cancer, and testicular cancer. In the past, it was thought that platinum-based compounds could not penetrate the blood-brain barrier (BBB). However, studies have shown that it can penetrate the BBB in small amounts, and neural populations including progenitor cells, oligodendrocytes, and hippocampal neurons are particularly vulnerable [14–16]. In vitro studies found that cisplatin increased hippocampal neuron apoptosis and reduced neurogenesis. It is also involved in inducing damage to mitochondrial DNA, which leads to mitochondrial dysfunction and increased oxidative stress. Neuronal integrity and cognitive function are eventually impaired [17]. Antitumor drug 5-fluorouracil (5-Fu) can decompose myelin sheath integrity and is associated with hippocampal neurodegenerative defects and mitochondrial dysfunction [18]. Rodents exposed to cyclophosphamide or doxorubicin for long periods of time show impairments in spatial memory and episodic memory, with disruption of hippocampal neurogenesis [19]. Intrathecal injection of methotrexate and cytarabine, as a treatment for acute lymphoblastic leukemia in children, has been shown to impair the dendritic architecture and hippocampal-dependent cognitive function in mice [20]. Methotrexate destroys the oligodendrocyte compartment and white matter integrity and is associated with cognitive impairment [21]. These all show a direct adverse effect of chemotherapy drugs on neurons in the brain.

### 2.2. Indirect Mechanisms of Chemotherapy Drugs

Patients receiving chemotherapy obviously have a higher risk of cognitive deficits but not necessarily due to neurotoxic effects. Cognitive disorders may also be caused by indirect mechanisms such as metabolic and hormonal abnormalities caused by treatment, activation of inflammatory cytokines, genetic polymorphisms, fatigue, damage to other body organs, or microvascular disease [22, 23].

#### 2.2.1. Cytokine Disorders

Chemotherapy drugs, the tumor itself, and the patient's long-term physiological and psychological stress can lead to increased production of inflammatory cytokines such as tumor necrosis factor alpha (TNF-*α*), interleukin 1 (IL-1), and interleukin 6 (IL-6). These cytokines entering the brain through the BBB causes a local inflammatory response in the brain, which also leads to the destruction of the structure and integrity of the BBB and epigenetic changes [24]. The study found that the volume of hippocampus in the breast cancer group receiving chemotherapy was relatively low and was associated with higher TNF-*α* levels and lower IL-6 levels, providing a basis for inflammatory cytokine-mediated CRCI [25]. In addition to hippocampal dysfunction, increased cytokines caused by chemotherapy appear to be associated with disruption of neuroplasticity [26]. Cheung et al. conducted a multicenter, prospective cohort study to verify the relationship between inflammatory cytokine levels and CRCI in breast cancer patients. They found that the increase in IL-1*β* and IL-6 was associated with a slower response rate and self-perceived cognitive impairment, while increased IL-4 concentration was related to improved response speed and cognitive function, which means that IL-4 has potential neuroprotective effects, consistent with the previous animal studies [27, 28]. Some scholars have proposed that, in addition to the nature of cytokines, the balance and/or ratio of the quantitative cytokine levels are also related to CRCI. They found TNF-*α* was implicated in the acute cognitive trajectory, while IL-6 and IL-8 were involved in the persistent cognitive trajectory [29]. Interestingly, high IL-8 levels have been reported to be positively correlated with memory and executive function in other studies [30, 31]. This may be related to chemotherapy regimentation and tests in the neuropsychological subfield.

#### 2.2.2. Oxidative Stress

There are many scholars to explore the relationship between oxidative stress and CRCI. Anthracyclines such as adriamycin are often used to treat hematological malignancies and solid tumors. After doxorubicin enters the brain, it promotes the occurrence of oxidative stress by destroying the mitochondria and inducing the peroxisomes, leading to cellular DNA damage and dysfunction [32]. Meanwhile, doxorubicin can induce the increase of TNF-*α* in the plasma and brain, resulting in the central nervous system toxicity. Studies have shown that patients receiving anthracycline chemotherapy show impaired speech memory and decreased functional connectivity in the left precuneus. Its mechanism is related to increasing mitochondrial dysfunction, inducing the oxidative stress and the release of proinflammatory cytokines [33]. Oxidized phosphatidyl choline levels in the cerebrospinal fluid increased during treatment with methotrexate in children with acute lymphoblastic leukemia, which can lead to a decline in cognitive functions such as executive function [34]. In addition, carmustine and cyclophosphamide are also involved in the process of nervous system oxidative stress [35].

#### 2.2.3. Genetic Polymorphism

There are several genetic polymorphisms related to the cognitive function. Apolipoprotein E (APOE) mainly plays a role in lipid metabolism. The *ε*4 allele of APOE is associated with neuronal repair and plasticity. There is evidence that *ε*4 allele of APOE increases the risk of cognitive decline in Alzheimer's disease [36]. Aged APOE4 knock-in mice after doxorubicin treatment showed an impairment of spatial memory and decreased the volume of gray matter in the frontal cortex [37]. Ahles and his colleagues performed neuropsychological tests on cancer patients undergoing chemotherapy and found that patients carrying the APOE*ε*4 allele showed worse visual memory, spatial abilities, and speed of mental movement than uncarriers. The APOE*ε*4 allele may be a potential genetic marker for increasing the vulnerability to cognitive impairment after chemotherapy [38]. But, a study about patients with testicular cancer found APOE4 did not increase the risk of cerebral white matter structure connectivity changes after chemotherapy [39]. Catechol-O-methyltransferase (COMT) catalyzes the breakdown of catecholamines through the methylation of catecholamine neurotransmitters such as dopamine, adrenaline, and norepinephrine, thereby regulating dopamine levels in the frontal cortex. Dopamine is involved in frontal cortex-mediated executive and memory functions. A study found an association between COMT gene polymorphisms and higher risk of CRCI in breast cancer survivors after chemotherapy [40]. The brain-derived neurotrophic factor (BDNF) is mainly expressed in the central nervous system, promoting the survival of nerve cells and increasing the synaptic plasticity and neurogenesis. Carriers of the BDNF Met allele reduce the occurrence of CRCI, especially in terms of speech fluency and multitasking [41]. Other genetic polymorphisms, such as those related to the folic acid pathway, estrogen receptor alpha genetic polymorphism, and DNA methyltransferase 1 polymorphism are also related to cognitive function [42–44]. In summary, genetic factors are associated with cognitive impairment in cancer patients after treatment.

#### 2.2.4. Hormone Level

Estrogen receptor modulators (SERMs) and aromatase inhibitors (AIs) are commonly used as adjuvant treatments for breast cancer. Similar to androgen deprivation therapy for prostate cancer, they can alter hormone levels in patients. Estrogen and testosterone have neuroprotective and antioxidant functions. Estrogen even has a certain effect on maintaining telomere length [45]. Therefore, reduced hormone levels may cause CRCI. Surprisingly, multiple studies have shown that impaired cognitive function areas such as speech memory, vocabulary fluency, and decision-making function appear after breast cancer patients undergo endocrine therapy [46–48], which may be related to the menopause period and timing of treatment [49]. Recently, Kathleen et al. conducted a six-year prospective longitudinal study and found no endocrine therapy to adversely affect the cognitive function of breast cancer survivors. Androgen deprivation therapy (ADT) for prostate cancer causes a decrease in the testosterone, which may be related to the cognitive effects of the hippocampus. Studies have found that language skills, short-term memory, psychological flexibility, and inhibitory control of prostate cancer patients receiving ADT are impaired [50], but a systematic review shows that the relationship between ADT and overall cognitive impairment in the overall analysis is inconclusive [8]. Obviously, there is still controversy in this area, and more detailed investigations are needed to further explore the relationship between hormone therapy and cognitive function in the future [51].

Studies in noncancer populations have shown that activation of the hypothalamus-pituitary-adrenal axis (HPA) plays an important role in learning and memory. Cancer patients with long-term stress stimulate the HPA axis to activate excessively, increasing the cortisol secretion, which triggers damage to the brain structure and function including hippocampus [52–54]. Research studies suggested that elevated cortisol levels in cancer patients could be predictors of cognitive impairment [55], but patients in this regimen did not receive chemotherapy. Toh et al. found that prechemotherapy levels of dehydroepiandrosterone (DHEA) and its sulfated form (DHEAS) were positively correlated with verbal fluency and mental acuity, but this study did not find a link between hormone levels and working memory and attention, inconsistent with the previous studies [56]. Future studies will also need to look at specific areas of cognitive impairment.

### 2.3. Other Possible Mechanisms

Exosomes, nanovesicles secreted by most types of cells, are released into the extracellular matrix after fusion of the outer membrane of the multivesicle and the cell membrane. They mainly play a role in intercellular signaling. Exosomes have the ability to cross the BBB and participate in the neuronal development, repair, maintenance, and regeneration in the nervous system under physiological conditions [57]. Some investigators pointed out that cancer and its treatment can lead to changes in exosome content that may affect neurobiological functions and behavior. Exosomes may not only directly regulate the cellular mechanism to affect brain activity and impair cognitive function but also promote the occurrence of CRCI by intensifying the destruction of BBB and mediating immune regulation [58].

In addition to the above, biological aging, cognitive reserve, fatigue, sleep, anemia, posttraumatic stress of cancer, DNA damage, and vascular factors all have certain effects on cognitive function [45, 59–61]. A research finds that age and cognitive reserve before treatment are related to CRCI, which are important indicators for predicting changes in processing speed after treatment [59]. Chemotherapy can accelerate age-related development of tauopathy, which leads to the loss of synaptic integrity and cognitive disorder [62]. Tumor-associated anemia causes cognitive impairment mainly by reducing the oxygenation in the brain, causing fatigue symptoms [63]. Chemotherapy drugs that affect the blood coagulation may cause endothelial dysfunction and then microbleeds, so Sepehry et al. hypothesized a causal relationship between chemotherapy-related microbleeds and chemotherapy-related cognitive disorders [61]. This hypothesis provides a new direction for improving cognitive impairment after chemotherapy in cancer patients.

## 3. Assessment Methods

There are currently no guidelines for the diagnosis of CRCI. There are three main ways to assess the cognitive function of cancer patients.

### 3.1. Self-Reported Cognitive Impairment

Cancer patients generally complain of changes in cognitive function after chemotherapy, affecting the quality of life. Commonly used assessment tools for self-reported cognitive function include the European Organization for Research and Treatment of Cancer QLQ-C30 (EORTC QLQ-C30), Functional Assessment of Cancer Therapy-Cognitive Function (FACT-COG) Questionnaire, Cognitive Failures Questionnaire (CFQ), Multiple Ability Self-report Questionnaire (MASQ), and Patient's Assessment of Own Functioning Inventory (PAOFI) [64]. It was reported changes in the hippocampal functional connectivity was related to the self-reported cognitive impairment [65]. A systematic review showed that most studies have shown a lack of correlation between self-reported cognitive symptoms and neuropsychological outcomes, while only a small percentage reported significant associations between the two in a limited cognitive domain. This may be because subjective cognitive function is greatly affected by symptoms of age, race, cognitive reserve, anxiety, fatigue, and depression, so we need more objective and accurate assessment methods [64, 66]. Dorajoo and his colleagues designed a web-based tool to predict CRCI with a sensitivity of 57.1% and specificity of 76.9% that could help clinicians with early screening [39].

### 3.2. Neuropsychological Tests

The clinical diagnosis of CRCI is usually determined through neuropsychological testing. Standard neuropsychological tests are more objective and accurate than subjective perception. The International Cognition and Cancer Task Force (ICCTF) recommends the Hopkins Verbal Learning Test-Revised (HVLT-R), Trail Making Test (TMT), and the Controlled Oral Word Association (COWA) of the Multilingual Aphasia Examination to quantify tumor-related cognitive impairment [67]. Cognitive impairment is generally considered to be two or more test scores equal to or lower than the population normative standard of −1.5 standard deviation and/or single test scores equal to or lower than the average −2.0 SDs. In addition to the above tools, the cognitive assessment tools used in clinical trials are relatively more diverse, and commonly used ones are Mini-Mental State Examination (MMSE), Montreal Cognitive Assessment Scale (MOCA), Wechsler Adult Intelligence Scale, California Verbal Learning Test-II, Revised Rey–Osterrieth Complex Figure Test, High-Sensitivity Cognitive Screen, and so on [68]. All in all, there is no universal CRCI-specific scale. It is critical to develop a comprehensive, fast, and accurate CRCI scale.

### 3.3. Neuroimaging Manifestations

Brain morphology and function also changed during chemotherapy. In order to improve the homogeneity of research methods in the fields of cancer and cognition, unified imaging methods are obviously very effective. So, ICCTF recommends using structural and functional magnetic resonance imaging (fMRI) and positron emission tomography (PET) to research the chemobrain [69].

MRI revealed morphological changes in the brain structure of patients after chemotherapy, such as insula, bilateral hippocampal gyrus, left anterior cingulate cortex, decreased gray matter volume, and damage to white matter integrity. The hippocampus and gray matter are related to attention, learning, memory and other cognitive functions [7, 60, 70, 71]. Other researchers who use the diffusion tensor imaging (DTI) have shown that impaired white matter integrity in breast cancer patients receiving adjuvant chemotherapy is associated with decreased cognitive functions such as attention and verbal memory [72, 73]. Amidi A and his colleagues conducted the first longitudinal study of the changes in the brain structure network of cancer patients and found that the brain structure network of testicular cancer patients based on cisplatin chemotherapy was broken and their cognition impaired [74]. But their research also has certain limitation, namely, the limitation of a relatively small sample size.

In addition to the changes in the brain structure, the functional connectivity of brain network was also affected after chemotherapy. Miao et al. found the functional connectivity of the anterior cingulate cortex was decreased in breast cancer patients after chemotherapy, and this connectivity was associated with executive function, which suggested that changes in functional connectivity might be the pathological basis of CRCI [75]. Another study examined the resting state functional MRI and Atlas analysis of 34 breast cancer survivors undergoing chemotherapy and found that the topology of global and regional brain networks characteristic of breast cancer that received or did not receive chemotherapy could be destroyed, compared with the healthy control group, resulting in an reduced efficiency of parallel information transmission [76]. The connectivity of hippocampal functional networks increased after chemotherapy in breast cancer patients, which was negatively correlated with prospective memory. It suggested maladaptive and/or pathogenic mechanisms in CRCI [77]. Some researchers have focused on the default mode network (DMN) as a potential biomarker for chemotherapy-related brain damage [78]. Brain areas of DMN include the posterior cingulate gyrus/Precuneus, medial prefrontal cortex (MPFC), bilateral angular gyrus (AG), bilateral lateral temporal cortex (LTC), and bilateral hippocampus [79]. It is involved in the implicit learning, autobiographical memory, searching and monitoring the external environment, and process of self-reflection. Impaired cognitive function in lung cancer patients undergoing chemotherapy is associated with reduced resting functional connectivity in DMN [80]. A study, however, showed that functional connection in the brain network of breast cancer survivors changed whether chemotherapy was used or not [81]. Some researchers have applied multivariate pattern analysis (MVPA) to DMN analysis, increasing the sensitivity of identifying cognitive impairments. Kesler et al. were the first to apply MVPA to chemotherapy-related cognitive dysfunction, which can accurately distinguish postchemo breast cancer patients, nonchemotherapy patients, and healthy controls with 90-91% accuracy [82].

In addition to the commonly used imaging techniques, the pulsed arterial spin labeling (PASL) MRI perfusion, magnetic resonance spectroscopy (MRS), and electroencephalography (EEG) are also used in CRCI [71, 83–85]. Because of the high price, neuroimaging methods are widely used in the study of CRCI. It provides different explanations for the mechanism of CRCI and also provides a new perspective for assessing the existence of CRCI and formulating clinical interventions.

## 4. Management

### 4.1. Nonpharmaceutical Therapy

Diet plays an important role in the brain structure and function. Previous studies about breast cancer suggested that the intake of fruits and vegetables was correlated with verbal fluency and executive function. Zuniga and Moran found that higher serum carotenoid concentrations might play a positive role in the cognitive development of breast cancer patients [86]. This may be related to its role as a reactive oxygen species scavenger in the brain, reducing the oxidative stress and inflammation. Some researchers also recommend that a diet rich in long chain, omega-3 fatty acids and low sugar content could protect neurons from the toxic effects of chemotherapy through preventing or reducing neuroinflammation and oxidative stress [87]. Therefore, adjusting the diet structure and providing targeted nutrition can provide the basis for the treatment of CRCI.

Carrying out physical and mental activity interventions and physical exercises such as yoga, qigong, and tai chi can improve cognitive problems related to cancer, which may be related to increasing cerebral blood flow, increasing neuroplasticity, promoting nerve formation, and reducing inflammatory factors [88–90]. Exercise intensity is medium or above, 2–5 times a week, and more than 8 weeks is better. Reid-Arndt et al. conducted tai chi training on 23 female cancer patients for 10 weeks and found that their neuropsychological functions including attention, memory, executive function, and processing speed were significantly improved [91]. A randomized placebo-controlled trial of the impact of high-intensity intermittent endurance training on CRCI in women with breast cancer is still in progress [92]. However, 50% of the neurons that increase in physical exercise experience programmed death within weeks before establishing a functional connection with other neurons. Studies have shown that cognitive training can extend the survival of neurons in the dentate gyrus to months. This training process involves associative learning, spatial learning, and even physical skills learning. There is a positive correlation between the training intensity and the number of surviving cells. That is to say, when the training task itself has a certain degree of difficulty, learning seems to have the greatest impact on neurogenesis [93, 94].

In addition to the above, cognitive behavioral therapy (CBT) can also help CRCI to some extent. CBT includes cognitive therapy and behavioral therapy. Cognitive therapy is mainly used to effectively correct the cognitive distortions through education, cognitive reconstruction, and role change, which can help eliminate the negative emotions and unsuitable behaviors and improve symptoms such as sleep quality and psychological stress. Behavior therapy is similar to cognitive training. It mainly encourages the patients to conduct relaxation training, role-playing, group activities, and behavioral blocking, so that they can learn the adaptive behavior through learning to reduce the impact of cognitive impairment on quality of life [95]. An article published in 2019 used a randomized controlled trial to explore the effects of computer-assisted cognitive rehabilitation on CRCI, and the result showed a positive effect, with a relative improvement in the quality of life of cancer patients [96]. Zeng et al. conducted a network meta-analysis of the ten nonpharmacological interventions for the CRCI in the non-central nervous system, which compared the effects of all interventions to provide more comprehensive evidence-based data. They eventually found that meditation, cognitive training, cognitive rehabilitation, and exercise interventions had statistically significant effects on CRCI [97].

### 4.2. Pharmaceutical Therapy

#### 4.2.1. Antidementia Drugs

There are no guidelines for the treatment of CRCI. A reversible acetylcholinesterase inhibitor donepezil is commonly used in the treatment of AD patients. A number of randomized controlled studies have found that donepezil has a positive effect on the cognitive function, especially speech memory, of cancer patients after chemotherapy. The reason may be that donepezil could increase the bioavailability of acetylcholinesterase, reduce the atrophy of the cholinergic system of the basal ganglia, and increase the cerebral perfusion [98, 99]. Memantine is one of the antidementia drugs as an *N*-methyl-D-aspartate receptor (NMDA) antagonist. Studies have shown that memantine reduces the possibility of cognitive impairment in patients with brain metastases after whole brain radiotherapy. Further clinical research about CRCI is still needed in the future [100].

#### 4.2.2. Central Nervous System Stimulant

Methylphenidate, a dopaminergic and noradrenergic agonist, is commonly used to treat attention deficit hyperactivity disorder in children. Escalante and colleagues found that, although methylphenidate did not improve cancer-related fatigue, patients' cognitive functions such as speech learning, memory, visual perception, and scanning speed were significantly improved [101]. But, the conclusion is still controversial. Results of randomized controlled trials by Mar Fan and Lower showed that cognitive function did not improve after cancer patients received methylphenidate or dextromethylphenidate [102, 103].

The central nervous system stimulant modafinil mainly acts on the hypothalamus sleep regulation center by reducing the gamma-aminobutyric acid (GABA) in the preoptic region that induces sleep. It may also have the effect of changing the CRCI of tumor patients. Kohli et al. found modafinil improved memory and attention in breast cancer survivors. But, their study included only patients who showed signs of fatigue, so it would be a bit odd to speculate that modafinil would work in all cancer patients [104].

#### 4.2.3. Other Potential Neuroprotective Drugs

CRCI has previously been reported to be associated with anemia, leading some researchers to suggest that improvements in hemoglobin levels may affect the cognitive function in cancer patients. But to date, the current indication for EPO in cancer is mainly anemia caused by chemotherapy [105]. No clinical studies have shown a clear neuroprotective effect of erythropoietin in patients undergoing chemotherapy. Ginkgo biloba is reported to improve the cognitive function in patients with AD. Attia et al. evaluated the cognitive function in patients with brain tumors undergoing radiotherapy after treatment with ginkgo biloba and found significant improvements in tests of speed, executive function, and memory. But, this trial did not design a randomized control group [106]. Correspondingly, Barton et al did not find a correlation between Ginkgo biloba and CRCI [107]. Nicotine is a nicotinic receptor agonist. Some researchers used transdermal nicotine transdermal patches or placebo patches for cancer patients with long-term chemotherapy and perform subjective cognitive function assessments and objective neuropsychological tests. The results showed that the self-reported cognitive symptoms of the subjects had greatly improved, but due to the large placebo effect and the limitations of the trial itself, investigators had not been able to determine whether there was a drug effect [108]. 2-Mercaptoethanesulfonate, *N*-acetylcysteine, or melatonin can prevent oxidative stress associated with chemotherapy and may also be a direction for CRCI prevention [109, 110]. Melatonin is a physiological sleep regulator secreted mainly by the pineal gland. A randomized, double-blinded, placebo-controlled trial demonstrated the neuroprotective effect of melatonin on the neuroplasticity process. It may offset the cognitive function decline, impaired sleep quality, and depression symptoms of breast cancer patients during adjuvant chemotherapy [111]. Antioxidant *N*-acetylcysteine alters the cognitive deficits mainly by reversing mitochondrial dysfunction, preventing free radical production, reducing hippocampal neuron apoptosis, and dendritic spine loss [112]. A study in mice found that the histone deacetylase 6 inhibitor, ACY-1215, improved established cisplatin-induced cognitive impairment by restoring mitochondrial and synaptic damage [113]. However, these relevant clinical trials are still lacking. Clinical trials of lithium, pioglitazone, ramipril, docosahexaenoic acid, ibuprofen, and other drugs for CRCI are also ongoing [114].

To sum up, there are no specific drugs that can prevent and treat CRCI. More large, reproducible experimental studies are needed in this area.

### 4.3. Others

Repeated transcranial magnetic stimulation (rTMS) can improve patients' cognitive function. rTMS is a noninvasive method of brain stimulation. It mainly regulates the excitability of the cerebral cortex, neuroplasticity, and production of neurotransmitters such as BDNF through a very short high-intensity magnetic field. Meanwhile, high-frequency rTMS can increase the brain blood flow in the stimulated area and promote the glucose metabolism [115]. Transcranial direct current stimulation (tDCS) also increases the ability of learning and cognition [116], but they are mainly used in the cognitive impairment caused by Alzheimer's disease (AD) and Parkinson's disease (PD). There is no clinical evidence for their application in the CRCI population.

Acupuncture can also improve CRCI. Tong et al. conducted traditional Chinese medicine acupuncture therapy on 40 breast cancer patients after chemotherapy and found that their cognitive function improved compared with that before treatment. The mechanism may be related to inducing the expression of serum BDNF and protecting neurons [117]. In another study, in addition to neuropsychological evaluation of cancer patients, MRI examinations were performed to obtain DTI and MRS data. The results showed that the neuropsychological performance of the subjects was improved after acupuncture, and the changes in DTI parameters and *N*-acetylaspartate (NAA) concentrations were lower than those in the control group. This indicates that the prevention or reduction of CRCI by acupuncture may be attributed to its reduction in demyelination and enhancement of hippocampal neurons vitality [118]. And, a recent double-blind randomized controlled trial showed that compared with minimum acupuncture stimulation, electroacupuncture trigeminal nerve stimulation plus body acupuncture showed advantages in improving the quality of life of cancer patients and reducing working memory impairment [119]. At the same time, antidepressants such as fluoxetine may alleviate CRCI by balancing cytokines [120]. Aspirin is known to have anti-inflammatory properties. Since inflammatory factors are involved in CRCI, can anti-inflammatory drugs be used to treat CRCI? The scientists tested this in mice and found that aspirin could prevent but not effectively reverse the cognitive damage [121]. Due to the small sample size of the current study, multicenter, large-sample randomized controlled clinical trials will be needed in the future to further prove the therapeutic effect and mechanism of these therapies.

## 5. Conclusion

Compared with the study of Wefel et al. in 2015, our review explores the pathogenesis of CRCI in greater depth. First of all, we have updated the study on the relationship between cytokines and CRCI, supplemented the effect of gene polymorphism such as DNA methyltransferase 1 polymorphism on cognitive impairment, presented the easily overlooked effect of HPA axis, and described a new perspective on the involvement of exosomes in cognitive impairment. It is a new topic, so researchers can explore it to provide new ideas for treatment options. Secondly, we have introduced three ways of CRCI diagnosis in detail, which are self-reported cognitive impairment, neuropsychological scale, and imaging findings. Finally, in terms of treatment strategies, nondrug therapy in addition to physical exercise and cognitive rehabilitation that have been described, we find that there have been researchers who have linked diet to CRCI. Optimal diet can have a positive effect on cognitive function. And, acupuncture also contributes to this effect. In addition, we have also introduced the research status of a variety of drugs including donepezil, memantine, methylphenidate, modafinil, ginkgo biloba, and melatonin. Some of them need more preclinical studies and large sample clinical studies to be further verified.

In conclusion, although the research on CRCI has made good progress so far, the existing research results are divergent and there are contradictory conclusions. Multiple mechanisms are involved in cognitive impairment in cancer patients, which refer to the direct neurotoxicity of chemotherapeutic drugs, mediated cytokine imbalance, oxidative stress, and changes in hormone levels. Fatigue, sleep, cognitive reserve, mental state, and patients' own factors also play significant roles in the generation of CRCI. The diagnosis of CRCI currently makes use of existing neuropsychological scales, and the sensitivity and specificity of the web-based tool need to be improved. Therefore, how to quickly identify and evaluate CRCI and maintain high specificity and sensitivity to help clinicians better predict and interfere with CRCI requires researchers to work together. In addition to the above, the development of targeted interventions to effectively prevent CRCI is also the key to improving the cognitive function and the quality of life of cancer survivors. However, most of the current studies are composed of small sample studies. It is necessary to conduct large-scale longitudinal studies to improve the reliability and accuracy in the future.
